# *Mycobacterium tuberculosis* suppresses protective Th17 responses during infection through multiple mechanisms

**DOI:** 10.1101/2025.05.08.652811

**Published:** 2025-05-13

**Authors:** Alex Zilinskas, Amir Balakhmet, Douglas Fox, Heyuan Michael Ni, Carolina Agudelo, Helia Samani, Sarah A. Stanley

**Affiliations:** 1. Division of Immunology and Molecular Medicine, Department of Molecular and Cell Biology, University of California, Berkeley, Berkeley, CA.; 2. Division of Infectious Disease and Vaccinology, School of Public Health, University of California, Berkeley, Berkeley, CA.

## Abstract

*Mycobacterium tuberculosis* (Mtb) causes more deaths annually than any other pathogen, yet an effective vaccine remains elusive. IFN-γ–producing CD4+ T cells are necessary but insufficient for immune protection. Data from humans shows that the development of IL-17a producing Th17 T cells correlates with protection against infection, however not all individuals develop a Th17 response. In mouse models, experimental vaccines can elicit protective Th17 cells, but Th17s are rare in primary infection. Here, we identify factors suppressing Th17 responses during primary Mtb infection. First, using Tbet deficient mice, we demonstrate that Mtb drives a Th1 response that is only partially protective and limits the production of protective Th17 cells in an IFN-γ independent manner. Next, we reveal that the ESX-1 type VII alternative secretion system in Mtb suppresses Th17 responses by inhibiting IL-23 production in dendritic cells. These findings define a new function of the ESX-1 secretion system in Mtb virulence, a long-standing question in tuberculosis research.

## Introduction

*Mycobacterium tuberculosis* (Mtb), the causative agent of tuberculosis (TB), remains one of the leading infectious disease killers worldwide, with 10.6 million new cases and 1.6 million deaths reported in 2021 (World Health Organization, 2022). The current vaccine, *Mycobacterium bovis bacillus Calmette-Guérin* (BCG), protects against childhood TB, but has highly variable efficacy, wanes over time, and provides minimal protection against adult pulmonary TB (Fine, 1995; World Health Organization, 2018; Kumar, 2021). Although there are 17 vaccine candidates currently in clinical trials, achieving robust vaccine elicited immunity remains a major challenge (Zhou and Zhang, 2023).

Interferon-γ (IFN-γ) produced by type 1 T helper (Th1) cells is essential for control of Mtb infection both in animal models and in humans (Flynn et al., 1993; Condos et al., 1998; Casanova and Abel, 2002). However, the MVA85A vaccine, despite inducing robust Th1 responses as intended, failed to provide protection against Mtb infection (Ndiaye et al., 2015). Patients with active TB can have high levels of IFN-γ in the lungs despite being unable to control infection (Ledesma et al., 2011). Thus, Th1 cells are necessary but not sufficient for controlling Mtb infection suggesting that other factors are important for effective immunity. Without a comprehensive understanding of which types and specific subsets of T helper cells essential to limit Mtb infection, developing an effective Mtb vaccine with consistent high efficacy will remain elusive.

Type 17 T helper (Th17) cells are known for their association with mucosal sites, including the lungs, where they have been shown to provide protection against several bacterial and fungal respiratory pathogens (Guglani and Khader, 2010). This protection relies on IL-17a production by Th17 cells (Gaffen, 2009). Many factors determine whether Th17 cells are elicited by infection. Antigen-presenting cells (APCs) drive the polarization of naïve T cells into Th17 cells by producing cytokines including IL-1β, IL-6, TGF-β, and IL-23. Activation of the RORγT transcription factor is essential for Th17 polarization (Lazarevic et al., 2011). There is also evidence that the route of infection with a bacterial pathogen influences whether a Th17 response is elicited. Infection with *Listeria monocytogenes* via the intranasal, but not intravenous route is required to observe a Th17 response to infection (Pepper et al., 2010). Th17 differentiation is opposed by Th1 differentiation via multiple mechanisms (Huber and Farrar, 2011). Tbet, the transcription factor essential for commitment to the Th1 lineage, has been shown to prevent Runx1 activation, thereby preventing Runx1-mediated transactivation of RORγT and therefore Th17 differentiation (Lazarevic et al., 2011). Additionally, IFN-γ, the signature cytokine of Th1s, inhibits Th17 differentiation and function via activation of STAT1, independent of Tbet (Yeh et al., 2014). Finally, production of type I IFN also can suppress Th17 differentiation (Zhang et al., 2011). Thus, several hallmarks of Mtb infection, namely strong Th1 responses, IFN-γ production, and type I interferon production tend to weigh against Th17s. Increasing evidence that Th17 cells correlate with protection against Mtb infection both in the non-human primate model (Gideon et al., 2015) and in humans (Perreau et al., 2013; Ogongo et al., 2021; Nathan et al., 2021; Sun et al., 2024) suggest that Th17 cells are an important component of protective immunity to Mtb. In mice, Th17 cells are rarely observed during primary Mtb infection, and the conditions under which Th17 cells are induced during infection remain unclear (Khader et al., 2007; Gopal et al., 2014; Segueni et al., 2016; Aguilo et al., 2016; Jong et al., 2022). However, experimental TB vaccines, particularly via a mucosal route, elicit Th17 cells that can confer protection against subsequent challenge in mice (Khader et al., 2007; Jong et al., 2022), demonstrating that Th17 cells can be protective against Mtb infection. Why Mtb infection does not robustly elicit Th17 cells during primary infection, despite causing infection at mucosal surfaces via airway infection, is unclear.

The ESX-1 type VII alternative secretion system is a fundamental determinant of Mtb virulence (Stanley et al., 2003). The ESX-1 secretion system has been demonstrated to subvert and/or manipulate immune responses, including by perforating phagosomal membranes, inducing detrimental host type I interferon, and inducing autophagy (Stanley et al., 2007; Simeone et al., 2012; Romagnoli et al., 2012). As much of the work examining ESX-1 function has been conducted in vitro, we lack a clear understanding of all the factors by which ESX-1 mutants are attenuated during in vivo infection. Interestingly, the Mtb lipid phthiocerol dimycocerosate (PDIM) is also required for virulence and has been linked to ESX-1 function through induction of type I IFN, which can suppress Th17 responses (Cox et al., 1999; Barczak et al., 2017). In addition, the ESX-1 secretion system was shown to suppress production of IL-12 p40 (Stanley et al., 2007), a subunit shared with IL-23, which promotes Th17 differentiation. These data suggest that Mtb virulence factors, including ESX-1 and PDIM may suppress the emergence of a protective Th17 response during Mtb infection.

Here we show that the induction of Th17 cells during Mtb infection is suppressed by multiple mechanisms. First, we show that the absence of a Th17 response during primary infection is not dependent on duration or route of infection. Instead, we show that mice lacking Tbet (*Tbx21*^−/−^) produce Th17 cells after Mtb infection, and that these cells provide IL-17a-dependent protection to early Mtb infection. The suppression of Th17 production by Th1 cells is independent of IFN-γ. Furthermore, we observe a strong Th17 response during infection with ΔESX-1 or PDIM-lacking Mtb in naïve mice, suggesting that these virulence factors function to suppress Th17 differentiation. Surprisingly, the ability of ESX-1/PDIM to suppress Th17 differentiation is not related to their enhanced induction of type I IFN. Instead, we show that during infection with these strains type 2 conventional dendritic cells (CD11b^+^ DCs, cDC2s), which are known to support Th17 differentiation, produce higher levels of IL-23 secretion in draining lymph nodes of Mtb infected mice (Schlitzer et al., 2013). These results demonstrate that Mtb utilizes the ESX-1 secretion system and PDIM to bias host T helper response towards Th1s and away from Th17s in a manner that limits but permits Mtb infection.

## Results

### Th17 induction during *M. tuberculosis* infection does not depend on duration or route of infection

**Figure 1.**
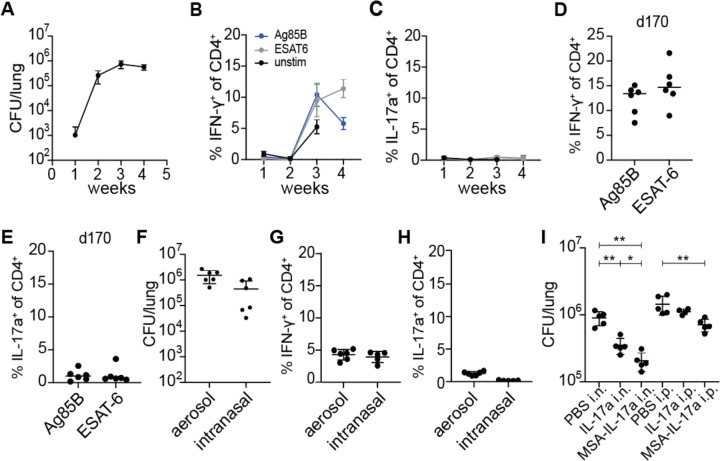
Lack of Th17 induction during WT *M. tuberculosis* infection is not due to duration or route of infection. **(A-C)** WT B6 mice were aerosol infected with WT *M. tuberculosis* Erdman strain and evaluated at 21 days post infection (dpi) for **(A)** bacterial burdens in lungs by CFU from lungs **(B)** IFN-γ^+^ CD4^+^ T cells and **(C)** IL-17a^+^ CD4^+^ T cells from lungs using flow cytometry. Cytokines were measured using ICS after restimulation with Ag85b or ESAT-6 peptide pools. **(D-E)** Mice were infected and analyzed at 170 dpi for **(D)** IFN-γ^+^ CD4^+^ T cells and **(E)** IL-17a^+^ CD4^+^ T cells in lungs using flow cytometry. **(F-H)** WT B6 mice were either aerosol infected or intranasally infected with WT *M. tuberculosis* Erdman strain and evaluated 28 dpi for **(F)** bacterial burdens in lungs by CFU, **(G)** IFN-γ^+^ CD4^+^ T cells and **(H)** IL-17a^+^ CD4^+^ T cells in lungs by flow cytometry. Cytokines were measured using ICS after restimulation with ESAT-6 peptide pool. **(I)** WT B6 mice were aerosol infected with WT *M. tuberculosis* and treated with PBS control, recombinant murine IL-17a (IL-17a) or recombinant murine IL-17a fused to murine serum albumin (MSA-IL-17a) daily either using the intranasal (i.n.) or intraperitoneal (i.p.) routes between days 11 to 20 post infection. CFU in lungs was measured at 21 dpi. Results are representative of at least 2 biological replicates. For all graphs, each dot represents an individual mouse. *p<0.05, **p<0.01 (unpaired nonparametric Mann-Whitney U test).

We first tested whether the duration or route of infection has an impact on the generation of Th17 cells during Mtb infection. To test for induction of Th17 cells, we infected wild type mice with ~100 CFU of WT *M. tuberculosis* via the aerosol route, a standard dose for the mouse model (Soldevilla et al., 2022). As previously published by multiple groups, we do not observe Th17 cells during the first month after Mtb infection of mice (Khader et al., 2007; Gopal et al., 2014; Segueni et al., 2016; Aguilo et al., 2016; Jong et al., 2022). At 4 weeks post infection, we observed the expected plateau of CFU in the lungs of infected mice indicating the onset of T cell-based control of infection (Figure 1A). With stimulation using peptide pools from model antigens ESAT-6 and Antigen 85B (Ag85B) (Andersen et al., 1995; Bekmurzayeva et al., 2013; Babaki et al., 2017), we observed only IFN-γ^+^ CD4^+^ (Th1) T cells (Figure 1B) and no IL-17a^+^ CD4^+^ (Th17) T cells at this or earlier timepoints (Figure 1C). Th17 cells have been observed in humans, at times presumably long after initial infection (Perreau et al., 2013; Ogongo et al., 2021; Nathan et al., 2021; Sun et al., 2024). We therefore tested whether Th17 cells arise at late timepoints after infection in mice. Mice continued to only have a Th1 response up to day 170 post infection (Figures 1D–E). It was previously reported that infection with the virulent HN878 “Beijing” strain of the L2 lineage resulted in induction of Th17 cells (Gopal et al., 2014). However, we did not observe a significant increase in IL-17a producing T cells in murine lungs when we infected with a Beijing strain at 3 weeks post infection (Supplementary Figure 1). The route of bacterial infection can impact which T helper cell types are induced. Infection via the intranasal route has been shown to elicit Th17 cells in response to infection with *Listeria monocytogenes* and Group A *Streptococcus* (Pepper et al., 2010; Dileepan et al., 2011). We therefore tested whether the intranasal route of infection results in a Th17 response in the lungs of Mtb infected mice. Mice were infected intranasally such that we inoculated ~100 CFU of WT Mtb into the lungs as measured on day 1 after infection. After four weeks of infection, mice had comparable levels of bacteria in the lungs between infection routes (Figure 1F). At this timepoint we again observed only IFN-γ producing CD4 T cells and very few IL-17a producing CD4 T cells in the lungs (Figures 1G–H). Thus, the route of infection with Mtb does not seem to impact the dominant Th1-biased response observed during primary infection of naïve mice with Mtb. We next investigated whether exogenous IL-17a treatment could provide protection against WT Mtb infection during primary infection. WT mice aerosol infected with WT Mtb were intranasally administered with either PBS control or 1 μg recombinant murine IL-17a daily between days 11 to 20 post infection for a total of 10 doses (Figure 1I). WT mice intranasally (i.n.) administered 1 μg recombinant murine IL-17a daily were slightly more protected with ~3-fold reduction in lung CFU compared to WT mice given vehicle control. However, we observed no effect of IL-17a when it was administered via the intraperitoneal (i.p.) route. Cytokines are known to have short half-lives in vivo. Fusing a cytokine to serum albumin can increase its half-life, permitting greater efficacy (Tan et al., 2022). We therefore fused IL-17a to murine serum albumin (MSA-IL-17a) and administered this construct either i.p. or i.n. at equimolar quantities compared to free IL-17a administered dosages. We found that i.n. delivery of this construct was more effective than delivery of IL-17a, with ~5-fold decrease in CFU observed in the lungs. In addition, fusion to albumin allowed us to see efficacy with i.p. administration, resulting in a ~2-fold decrease in lung CFU. These results indicate that IL-17a can induce protective immunity to Mtb in the context of a primary infection, and that this may have potential as a therapeutic approach.

### *Tbet*^−/−^ mice are protected by a robust Th17 response during early *M. tuberculosis* infection

**Figure 2.**
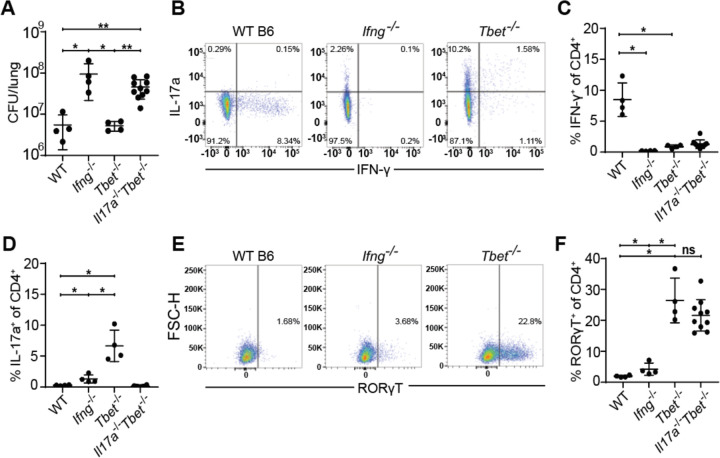
*Tbet*^−/−^ mice are protected from susceptibility to Mtb infection by the emergence of Th17 cells. WT B6, *Ifng*^*−/−*^, *Tbet*^*−/−*^, and *Tbet*^*−/−*^*Il17a*^*−/−*^ mice were aerosol infected with WT *M. tuberculosis* Erdman strain. *Tbet*^*−/−*^ and *Tbet*^*−/−*^*Il17a*^*−/−*^ mice were littermate-controlled. Mice were infected with Mtb and evaluated 21 days post infection for **(A)** CFU in the lungs of indicated genotypes or **(B-F)** cellular immune responses during flow cytometry. CD4^+^ T cells were gated on single, live, MHC-II^−^, Ly6G^−^, CD3^+^, γδTCR^−^, CD4^+^, CD8a^−^ cells for analysis of IFN-γ **(B,C)**, IL-17 **(B,D)** and RORγT **(E,F)**. Results in B-E are representative of 3 independent experiments. *, p < 0.05; **, p < 0.01 (unpaired nonparametric Mann-Whitney U test).

Because Th1 responses are known to inhibit Th17 responses (Lazarevic et al., 2011; Yeh et al., 2014), we next tested whether Th1 differentiation masks a protective Th17 response during Mtb infection. *Tbx21*, also referred as Tbet, encodes the essential transcription factor that drives the Th1 lineage (Lazarevic et al., 2011). Interestingly, *Tbet*^−/−^ mice lack Th1 T cells but are not as susceptible to infection with Mtb as are *Ifng*^*−/−*^ mice (Sullivan et al., 2005; Flynn et al., 1993). As expected, we found that *Ifng*^−/−^ mice were extremely susceptible to infection with Mtb, however *Tbet*^−/−^ had CFU in the lungs equivalent to wild type mice at 21 days after infection (Figure 2A). Only WT mice had IFN-γ producing CD4 T cells in the lungs at this timepoint (Figures 2B, 2C). Importantly, *Tbet*^−/−^ mice exhibited a robust population of IL-17a producing CD4 T cells at 3 weeks post infection, confirming that Tbet prevents Th17 differentiation during Mtb infection (Figures 2B, 2D). Importantly, IFN-γ deficient mice did not exhibit elevated levels of IL-17a producing CD4 T cells demonstrating that IFN-γ production is not the mechanism by which Th1 T cells limit a Th17 response during Mtb infection (Figure 2D).

A previous study demonstrated that Tbet constrains effective T cell responses by promoting the differentiation of terminally differentiated, non-protective intravascular Th1 T cells (Sallin et al., 2017). This study also used antibody blockade of IL-17a in Tbet deficient mice to conclude that IL-17a provides minimal protection in this setting (Sallin et al., 2017). However, given that antibody blockade is limited by its short duration and possibly incomplete inhibition, we tested whether genetically ablating IL-17a production in Tbet mice enhanced their susceptibility to Mtb infection. We therefore infected *Tbet*^−/−^ and *Tbet*^−/−^*Il17a*^−/−^ (*Il17a*^*icre/icre*^ referred to as *Il17a*^−/−^) littermate-controlled mice with Mtb and measured CFU and T cell responses in the lungs after 3 weeks of infection. *Tbet*^−/−^*Il17a*^−/−^ mice were highly susceptible to Mtb infection with a 10-fold increase in lung CFU compared to WT and *Tbet*^−/−^ mice, similar to the susceptibility observed in *Ifng*^*−/−*^ mice (Figure 2A). *Tbet*^−/−^*Il17a*^−/−^ mice completely lacked both IFN-γ producing (Figure 2C) and IL-17a producing CD4 T cells (Figure 2D). Notably, *Tbet*^*−/−*^*Il17a*^*−/−*^ mice still generated Th17 cells, as evidenced by the presence of the Th17 master transcription factor RORγT (Lazarevic et al., 2011), despite their inability to produce IL-17a (Figures 2E, 2F). Together, these results indicate that IL-17a production by Th17 cells confers significant protection against Mtb infection in *Tbet*^*−/−*^ mice, at least during early stages of infection.

### *M. tuberculosis* utilizes ESX-1 and PDIM to limit Th17 response in mice

**Figure 3.**
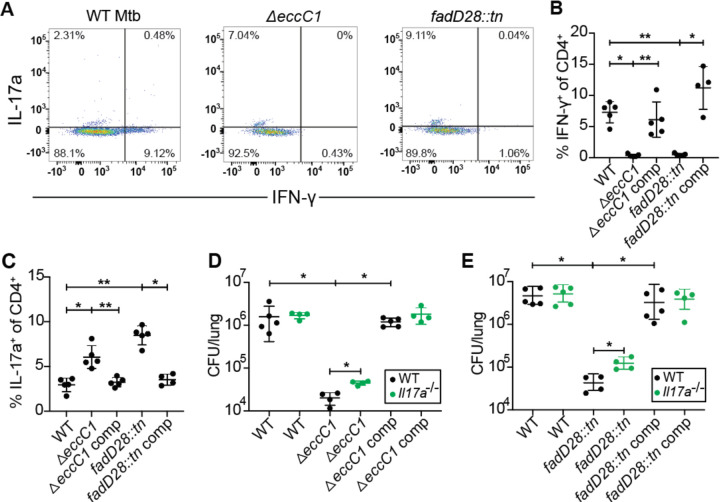
*M. tuberculosis* ESX-1 and PDIM virulence factors suppress Th17 response in mice. WT B6 mice were aerosol infected with indicated strains and evaluated at 21 days post infection. **(A)** Representative flow cytometry plots (cells gated on single, live, MHC-II^−^, Ly6G^−^, CD3^+^, γδTCR^−^, CD4^+^, CD8a^−^) of IFN-γ^+^ and IL-17a^+^ CD4^+^ T cell populations. 21 days post infection, mice were sacrificed and evaluated ESAT-6 peptide pool stimulated lung homogenates for **(B)** IFN-γ^+^ CD4^+^ T cells and **(C)** IL-17a^+^ CD4^+^ T cells by flow cytometry. **(E)** Littermate-controlled WT B6 and *Il17a*^*−/−*^ mice were aerosol infected with indicated strains at 21 days post infection with lung homogenate plated for CFU. Results are representative of 2 independent experiments. *, p < 0.05; **, p < 0.01 (unpaired nonparametric Mann-Whitney U test).

We next examined whether Mtb utilizes virulence factors to alter the host T helper response. The ESX-1 secretion system and the complex lipid PDIM are both known to influence production of cytokines known to impact Th17 differentiation (Stanley et al., 2007; Barczak et al., 2017; Hinman et al., 2021). Furthermore, these systems have been proposed to operate in the same virulence promoting pathway (Barczak et al., 2017; Augenstreich et al., 2017). We therefore tested whether ESX-1 and PDIM might promote virulence in part by suppressing Th17 differentiation (Figure 3A). To test for a role of the ESX-1 secretion system, we infected mice with Δ*eccC1,* which lacks an ATPase required for ESX-1 function (ΔESX-1) (Crosskey et al., 2020) (Supplementary Figure 2). We also utilized a mutant with an inactivating transposon insertion in *fadD28*, a gene required for PDIM biosynthesis (*fadD28::tn*, PDIM-lacking) (Cox et al., 1999) (Supplementary Figure 2). As expected, ΔESX-1 and PDIM-lacking mutants were highly attenuated during infection at 3 weeks, exhibiting a 100-fold reduction in bacterial burden while complemented strains had similar CFU as WT Mtb (Supplementary Figure 3). Interestingly, ESAT-6 peptide pool stimulation of lung cells in ΔESX-1 and PDIM-lacking Mtb infected mice had an ~5-fold reduction in %IFN-γ^+^ of CD4^+^ T cells (Th1s) (Figure 3B) and ~2-fold increase in %IL-17a^+^ of CD4^+^ T cells (Th17s) (Figure 3C) compared to complements of either mutant or WT Mtb. Similar results were seen with Ag85b peptide pool stimulation (Supplementary Figure 4). We tested if the induced Th17s are providing protection mediated by IL-17a by infecting littermate-controlled WT B6 and *Il17a*^−/−^ mice with either WT, ΔESX-1, or complemented ΔESX-1 Mtb, or either WT, PDIM-lacking, or complemented PDIM-lacking Mtb. Under the conditions where Th17s are highly induced, mice infected with either ΔESX-1 or PDIM-lacking Mtb, the *Il17a*^−/−^ mice had ~3–5 fold higher CFU than WT mice (Figures 3D, 3E). These results indicate that Mtb utilizes ESX-1 and PDIM to prevent the induction of a Th17 response that enhances protection against Mtb infection.

### Type I interferons boost Th1 induction and do not impact Th17 induction

**Figure 4.**
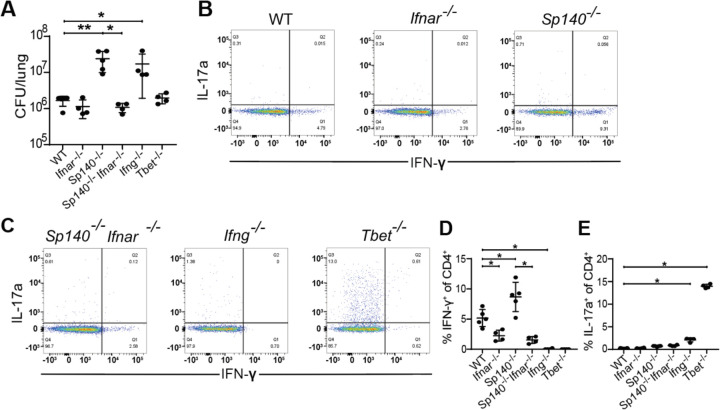
Type I interferon signaling boosts Th1 induction and does not impact Th17 induction. WT B6, *Ifnar*^*−/−*^, *Sp140*^*−/−*^, *Sp140*^*−/−*^*Ifnar*^*−/−*^, *Ifng*^*−/−*^, and *Tbet*^*−/−*^ mice were aerosol infected with WT *M. tuberculosis* Erdman strain. **(B, C)** Representative flow cytometry plots (cells gated on single, live, MHC-II^−^, Ly6G^−^, CD3^+^, γδTCR^−^, CD4^+^, CD8a^−^) of IFN-γ^+^ and IL-17a^+^ CD4^+^ T cells of these populations. 21 days post infection, lung homogenates were measured for **(A)** CFU and analyzed via flow cytometry for **(D)** IFN-γ^+^ CD4^+^ T cells and **(E)** IL-17a^+^ CD4^+^ T cells. Results in A, D, and E are representative of 2 independent experiments. *, p < 0.05; **, p < 0.01 (unpaired nonparametric Mann-Whitney U test).

As ESX-1 and PDIM are responsible for the host type I interferon response to Mtb infection (Stanley et al., 2007; Barczak et al., 2017), and type I IFN is known to suppress Th17 responses (Zhang et al., 2011), we investigated the impact of type I interferon signaling on Th17 induction during Mtb infection. We examined the effect of type I IFN both in WT B6 mice, and in *Sp140*^*−/−*^ mice that are highly susceptible to infection because of an excessive type I IFN response to infection (Ji et al., 2019). To abrogate type I IFN in each background we used mice deficient for the type I IFN receptor IFNAR. WT, *Ifnar1*^−/−^, *Sp140*^−/−^, *Sp140*^−/−^*Ifnar1*^−/−^, *Ifng*^−/−^, and *Tbet*^−/−^ mice were aerosol infected with WT Mtb. As expected, both *Sp140*^−/−^ and *Ifng*^−/−^ mice had up to 10-fold higher CFU than WT mice while *Ifnar1*^−/−^, *Sp140*^−/−^*Ifnar1*^−/−^, and *Tbet*^−/−^ mice were as resistant as WT mice at 3 weeks post infection (Figure 4A). *Sp140*^−/−^ mice had an almost 2-fold increase in %IFN-γ^+^ of CD4^+^ (Th1) T cells compared to WT mice, *Ifnar1*^−/−^ had a 2-fold reduction in %IFN-γ^+^ of CD4^+^ (Th1) T cells compared to WT mice, *Sp140*^−/−^*Ifnar1*^−/−^ mice had a slightly stronger 4-fold reduction in %IFN-γ^+^ of CD4^+^ (Th1) T cells compared to *Sp140*^−/−^ mice (Figures 4B–D). Importantly, deletion of IFNAR had no impact on Th17 induction in either WT or *Sp140*^−/−^ mice, although we observed the expected increase in Th17 cells in Tbet deficient animals (Figure 4E). These results indicate that type I interferon signaling only impacts the induction of Th1s with positive correlation while type I interferon signaling does not influence Th17 induction, suggesting that induction of type I IFN by ESX-1/PDIM is not the mechanism by which these virulence factors suppress Th17 differentiation.

### ESX-1 and PDIM alter conventional dendritic cell cytokine signaling in the mediastinal lymph node

**Figure 5.**
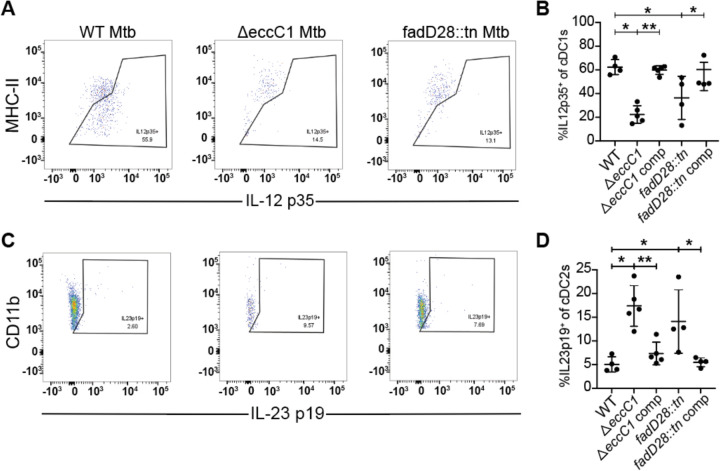
ESX-1 and PDIM alter conventional dendritic cell cytokine signaling in the mediastinal lymph node. WT B6 mice were aerosol infected with indicated strains and analyzed at 21 days post infection **(A,C)** Flow cytometry schematic for gating of **(A)** cDC1 (gated on single, live, CD3^−^, CD19^−^, CD11c^+^, MHC-II^+^, XCR1^+^, CD11b^−^) and **(B)** cDC2 (gated on single, live, CD3^−^, CD19^−^, CD11c^+^, MHC-II^+^, XCR1^−^, CD11b^+^) populations of mediastinal lymph nodes with representative flow cytometry plots of IL-12 p35^+^ and IL-23 p19^+^ gating of cDC1 and cDC2 populations respectively. 21 days post infection, mediastinal lymph nodes from WT B6 mice were aerosol infected with either WT, ΔeccC1 (ΔESX-1), complemented ΔeccC1::eccC1, transposon-inserted fadD28::tn (PDIM-lacking),or complemented transposon-inserted fadD28::tn + pfadD28 *M. tuberculosis* Erdman strain were extracted and analyzed for IL-12 p35^+^ cDC1s **(C)** and IL-23 p19^+^ cDC2s **(D)** by flow cytometry. Results in B and D are representative of 2 independent experiments. *, p < 0.05; **, p < 0.01 (unpaired nonparametric Mann-Whitney U test).

Previous work suggested that ESX-1 mutants fail to suppress IL-12 p40, a subunit shared between IL-12 and IL-23, expression from infected macrophages in vitro (Stanley et al., 2007). Because IL-23 promotes Th17 responses, and ESX-1 and PDIM are responsible for the lack of a Th17 response during Mtb infection, we next investigated if ESX-1 and PDIM suppress IL-23 production by dendritic cells during in vivo infection. We infected mice with ~100 CFU via the aerosol route and harvested mediastinal lymph nodes at 21 days post infection. We examined IL-12 and IL-23 production by conventional dendritic cells using ICS. To clearly distinguish between levels of these two cytokines we used ICS for the cytokine specific subunits: IL-12 p35 and IL-23 p19. First, we found that type 1 conventional dendritic cells (cDC1s), known to be specialized for IL-12 production, produce robust levels of IL-12 p35 during infection with WT Mtb (Figures 5A, 5B). However, both ESX-1 and PDIM deficient strains elicited significantly lower levels of IL-12 p35 from these cells (Figures 5A, 5B), consistent with the lower bacterial burdens in the lungs of mice infected with these mutants (Supplementary Figure 3). During infection with wild-type bacteria, we observed very little IL-23 p19 production by type 2 conventional dendritic cells (cDC2s) in the draining mediastinal lymph node (Figures 5C, 5D). Importantly, ESX-1 and PDIM-lacking Mtb infection resulted in a dramatic increase in the percentage of cDC2s that produce IL-23 p19 in mediastinal LNs (Figures 5C, 5D). We saw very little to no IL-23 p19 expression in cDC1s and IL-12 p35 expression in cDC2s with no differences between WT or ESX-1 and PDIM deficient strains (Supplementary Figure 5). These results indicate that the ESX-1 and PDIM virulence factors impact naïve T cell differentiation at the draining mediastinal lymph node by promoting IL-12, a Th1-polarizing cytokine, and limiting IL-23, a Th17-polarizing cytokine, expression in cDC1s and cDC2s respectively.

## Discussion

Immunity against *M. tuberculosis* (Mtb) requires T helper cell responses and IFN-γ as demonstrated by susceptibility of *Rag1*^−/−^ (Lai et al., 2024) and *Ifng*^−/−^ (Flynn et al., 1993) mice, and high co-morbidity between tuberculosis and low CD4 T helper cell counts in humans with AIDS (Amelio et al., 2019; Geremew et al., 2020). The requirement of a highly efficacious Mtb vaccine that elicits a T helper response strong enough to provide protection against Mtb infection remains desired. The MVA85A vaccine candidate elicited a strong Th1 response yet failed to provide protection against Mtb infection (Ndiaye et al., 2015) illustrating that a strong Th1 response is not enough to control Mtb infection. The development of a highly efficacious Mtb vaccine requires a rigorous understanding of which T helper cell types and subsets provide protection against Mtb infection. The recent reports of Th17s correlating to protection against Mtb infection in non-human primates (Gideon et al., 2015) and humans (Perreau et al., 2013; Ogongo et al., 2021; Nathan et al., 2021; Sun et al., 2024) suggest a protective role for Th17s in controlling Mtb infection. Herein, we showed that Th17s can provide protection that is IL-17a-dependent in mice, Mtb’s ESX-1 secretion system and PDIM enhance infection at least in part by preventing a Th17 response in mice, and exogenous IL-17a is protective against Mtb infection.

We have previously reported that infection in unvaccinated mice does not elicit a Th17 response, only a Th1 response, and that an intranasally administered cyclic dinucleotide adjuvant Mtb protein subunit vaccine elicits enhanced protection to Mtb infection in mice mediated by Th17s and in-particular IL-17a (Van Dis et al., 2018; Jong et al., 2022). There have been conflicting reports about Th17s and in-particular IL-17a providing protection against Mtb infection in the murine model of tuberculosis (Khader et al., 2007; Gopal et al., 2014; Segueni et al., 2016; Aguilo et al., 2016; Jong et al., 2022). Here, we report that Mtb prevents induction of Th17s during primary infection via the virulence factors ESX-1 and PDIM. Others have shown that intranasal vaccination of BCG, which lacks a functional ESX-1, also induced Th17s that provide protection against Mtb infection (Aguilo et al., 2016).

ESX-1 and PDIM have both been shown to be required for the permeabilization of *M. tuberculosis*-containing phagosomes and therefore induce the type I interferon response (Barczak et al., 2017; Stanley et al., 2007; Augenstreich et al., 2017; Watson et al., 2015). Thus, we investigated how impactful the type I interferon response is on the induction of Th1s and Th17s during WT *M. tuberculosis* infection. We found that the type I interferon response boosts the magnitude of the Th1 response but does not impact the induction of Th17s during *M. tuberculosis* infection. Additionally, we show that ESX-1 and PDIM promote IL-12 and limit IL-23 expression in conventional dendritic cells in the draining mediastinal lymph node during *M. tuberculosis* infection. Future work will focus on which immune pathways impacted by ESX-1 and PDIM gate-keep the induction of Th1 vs Th17 responses.

One possible answer for why humans induce Th1s and Th17s to Mtb infection while mice only induce Th1s might be because humans evolved cDC2s capable of expressing Th1-polarizing cytokine IL-12 and Th17-polarizing cytokines IL-1β, IL-6, and IL-23 (Rojas et al., 2017), and mice have evolved cDC2s incapable of IL-12 expression but still capable of expressing Th17-polarizing cytokines (Schlitzer et al., 2013).

A method of reliably inducing Th17s in mice during wild-type Mtb infection needs to be established to effectively study the role of Th17-mediated protection. Mice lacking Tbet (as known as *Tbx21*) respond to WT Mtb infection with a Th17 response and provide protection mediated by IL-17a during early infection. *Tbet*^−/−^ mice can be used as a model to effectively study how Th17s can provide protection against early Mtb infection in the absence of Th1s. We show that exogenous IL-17a treatment promotes control of Mtb infection indicating that there are some cells capable of sensing IL-17a and responding in a beneficial way for the host. The mechanisms by which IL-17a signaling promotes clearance of Mtb remains unknown. High Th17 plasticity permits Th17s to express a wide variety of T helper subset profiles and can range from pro-inflammatory Th17.1s that co-express IFN-γ and IL-17a to anti-inflammatory Tr1-like Th17s that co-express IL-10 and IL-17a (Stadhouders et al., 2018). Moving forward, it will be important to delineate in depth which Th17 subsets and which mechanisms downstream of IL-17 receptor signaling are important for controlling Mtb infection.

We have shown that Mtb utilizes the ESX-1 secretion system and lipid virulence factor PDIM to prevent Th17 induction during infection. Furthermore, ESX-1 and PDIM promotes expression of Th1-polarizing cytokine IL-12 in cDC1s and limits expression of Th17-polarizing cytokine IL-23 in cDC2s in the draining mediastinal lymph node. The type I interferon response induced by ESX-1 and PDIM only boosts Th1 induction and does not impact Th17 induction. Additionally, we have developed a model of inducing Th17s in mice (*Tbet*^−/−^ mice) during early Mtb infection which provide protection via IL-17a. And lastly, exogenous IL-17a treatment provides protection against Mtb infection. With this information, our understanding of how Mtb alters the host immune response to limit control of infection, and our development of models for studying Th17-mediated protection against Mtb infection provide insights to guide future Mtb vaccine design strategies.

## Materials and Methods

### Ethics statement

All procedures involved with the use of mice were approved by the University of California, Berkeley’s Institutional Animal Care and Use Committee (protocol 2015-09-7979). All protocols conform to federal regulations, the National Research Council Guide for the Care and Use of Laboratory Animals, and the Public Health Service Policy on Humane Care and Use of Laboratory Animals.

### Bacterial Culture

*Mycobacterium tuberculosis* strain Erdman was grown in Middlebrook 7H9 liquid medium supplemented with 10% oleic acid, albumin, dextrose, and catalase (OADC) (BD 212351), 0.4% glycerol, and 0.05% Tween 80 or on solid 7H10 agar plates supplemented with 10% OADC and 0.4% glycerol. Frozen stocks of *M. tuberculosis* were made from a single culture and used for all experiments.

### Mice

C57BL/6J (no. 000664), *Ifng*^−/−^ (B6.129S7-*Ifng*^tm1Ts^/J, no. 002287), *Tbet*^−/−^ (*Tbx21*^−/−^, B6.129S6-*Tbx21*^tm1Glm^/J, no. 004648), and *Il17a*^icre/icre^ (*Il17a*^−/−^, B6.129(SJL)-*Il17a*^tm1.1(icre)Stck^/J, no. 035717) mice were obtained from The Jackson Laboratory and bred in-house. *Ifnar1*^−/−^, *Sp140*^−/−^, and *Sp140*^−/−^*Ifnar1*^−/−^ mice were obtained from the Vance laboratory (UC Berkeley) and bred in-house. Mice were age matched for all experiments.

### Aerosol challenge experiments with *M. tuberculosis*

Mice between the age of 8 to 12 weeks old were infected via the aerosol route with *M. tuberculosis* strain Erdman using a nebulizer and full-body inhalation exposure system (Glas-Col, Terre Haute, IN). A total of 9 mL of diluted aerosol stock in MiiliQ water was loaded into the nebulizer to deliver 30–100 bacteria per mouse as measured by whole lung CFU 1 day post infection.

### Intranasal challenge experiments with *M. tuberculosis*

Mice between the age of 8 to 12 weeks old were infected with 40 uL of *M. tuberculosis* strain Erdman diluted in PBS administration for mice to inhale 30–100 bacteria per mouse as measured by whole lung CFU 1 day post infection.

### Flow Cytometry

Whole lungs were homogenized using GentleMACS C tubes (Miltenyi Biotec 130-093-237) in complete RPMI-1640 media supplemented with 10% FBS, 1 mM sodium pyruvate (Sigma Aldrich S8636), 0.01 mM HEPES, 1% Glutamax (Thermo Scientific 35050061), 1% non-essential amino acids (Gibco 11140–050), and 55 μM 2-mercaptoethanol (Gibco 21985–023) containing Liberase TM (Sigma Aldrich 05401119001) and DNase I (Sigma Aldrich 11284932001). Cells were stained with live/dead stain (ThermoFisher L23101), Fc block (Biolegend 101320), and surface stained with antibodies for CD3, Ly6G, CD4 (BD 740268, 551460, and 569182 respectively), MHC-II, pan-γδTCR, and CD8a (Biolegend 107605, 118124, and 100714 respectively) in Brilliant stain buffer (BD 566349). Cells were fixed and permeabilized with Foxp3 staining kit (Invitrogen 00-5523-00) before intracellular staining for RORγT, Foxp3 (BD 564723 and 562996 respectively), Tbet, IFN-γ, and IL-17a (Biolegend 644817, 505814, and 506903 respectively). Data were collected using a BD Symphony A3 flow cytometer (BD) and analyzed using FlowJo Software (BD).

Mediastinal lymph nodes were homogenized using complete RPMI-1640 containing Liberase TM (Sigma Aldrich 05401119001) and DNase I (Sigma Aldrich 11284932001) with sterile plunger of 3 mL syringe to pass dissociated tissue through 100 micron cell strainer. Cells were stained with live/dead stain (ThermoFisher L34966), Fc block (Biolegend 101320) and surface stained with antibodies for XCR1, CD11b, CD11c, Ly6G, MHC-II, CD3, CD19 (Biolegend 148220, 101261, 117318, 127627, 107606, 100233, and 115545 respectively) and Ly6C and CD64 (BD 755198 and 569507 respectively) in Brillant stain buffer (BD 566349). Cells were fixed and permeabilized with Foxp3 staining kit (Invitrogen 00-5523-00) before intracellular staining for IL-12 p35 (Invitrogen MA5–23559) and IL-23 p19 (BD 565317). Data were collected using a BD Symphony A3 flow cytometer (BD) and analyzed using FlowJo Software (BD).

### Exogeneous IL-17a administration

Mice were intranasally administered 1 μg endotoxin-free recombinant murine IL-17a (R&D systems, 7956-ML-100/CF) in 40 μL sterile PBS daily or 40 μL of vehicle control daily, or intraperitoneally administered 2 μg endotoxin-free recombinant murine IL-17a in 100 μL sterile PBS daily or 100 uL of vehicle control dailiy from days 11 to 20 post aerosol *M. tuberculosis* challenge.

MSA-IL-17a was generated by fusing a (GGGGS)3 linker between the C-terminus of mature murine serum albumin and N-terminus of mature murine IL-17a. MSA-IL-17a was purchased from Genscript USA, Inc. in endotoxin-free pH 7.2 PBS. Mice were intranasally administered 5.54 μg MSA-IL-17a (equimolar to 1 μg recombinant murine IL-17a) in 40 μL sterile PBS daily or 40 μL vehicle control, or intraperitoneally administered 11.09 μg MSA-IL-17a (equimolar to 2 μg recombinant murine IL-17a) in 100 μL PBS or 100 μL vehicle control daily between days 11 to 20 post aerosol *M. tuberculosis* challenge.

### Statistical Analysis

All data was analyzed using FlowJo v10 (FlowJo, LCC) and/or Prism 10 (GraphPad Software). All graphs were analyzed using Mann-Whitney U-test. Significant values are symbolized with asterisks of * < 0.05, ** < 0.01, and ns for non-significant.

## Supplementary Material

Supplement 1
