# Supplementary material for: Mycobacterium tuberculosis suppresses protective Th17 responses during infection through multiple mechanisms
Source: bioRxiv. 2025 May 13:2025.05.08.652811. Preprint. [Version 1] doi: 10.1101/2025.05.08.652811 (PMC12132438; doi:10.1101/2025.05.08.652811)
Supplement: Supplementary file 1 [file NIHPP2025.05.08.652811v1-supplement-1.pdf]

bioRxiv preprint doi: https://doi.org/10.1101/2025.05.08.652811; this version posted May 13, 2025. The copyright holder for this preprint (which was not certified by peer review) is the author/funder, who has granted bioRxiv a license to display the preprint in perpetuity. It is made available under aCC-BY 4.0 International license.

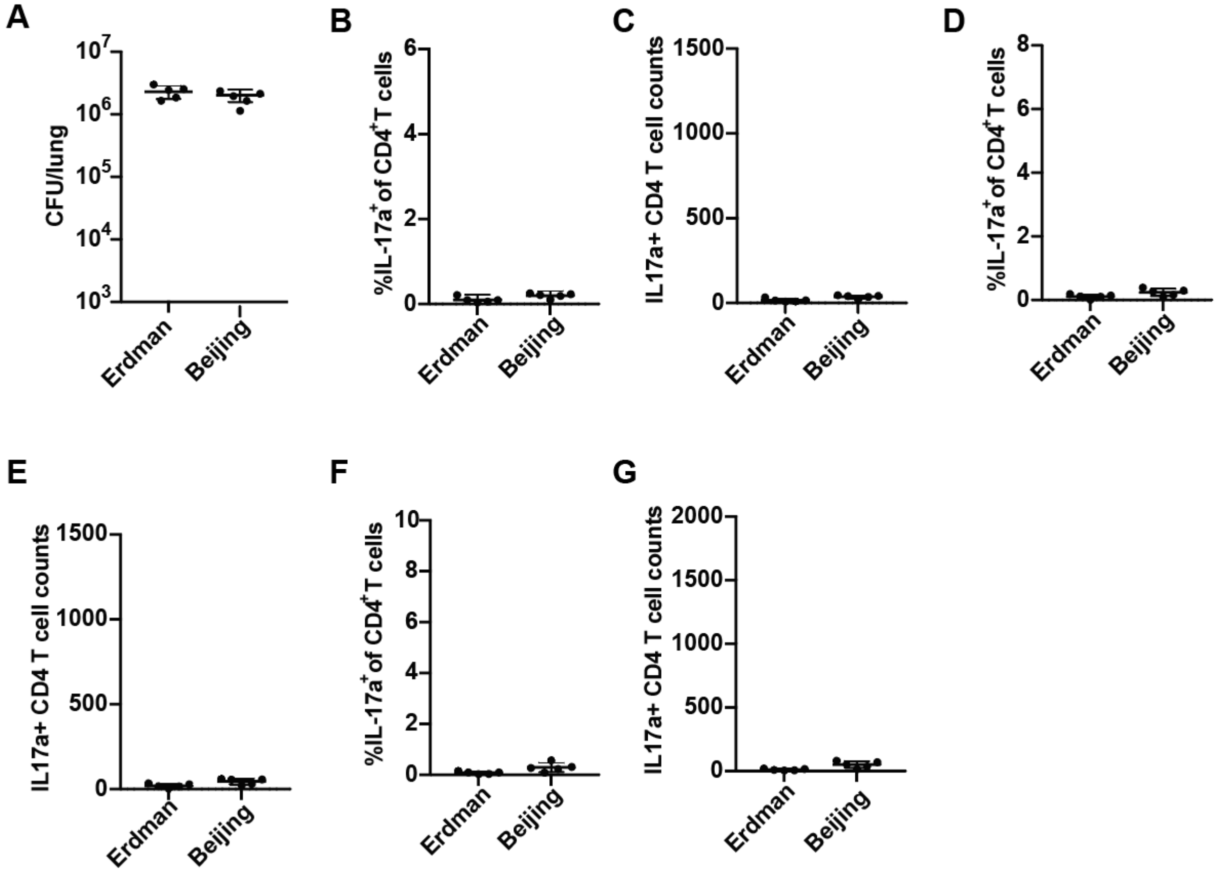

Figure S1. Mice were infected with either the Erdman or Beijing strain of Mtb via the aerosol route and evaluated at 21dpi for **(A)** CFU in the lungs or **(B-G)** IL-17a$^+$ CD4$^+$ T cells in the lungs. **(B,C)** unstimulated, **(D,E)** restimulated with Antigen 85B peptide, **(F,G)** restimulated with ESAT-6 peptide. Representative experiment of 2.

bioRxiv preprint doi: https://doi.org/10.1101/2025.05.08.652811; this version posted May 13, 2025. The copyright holder for this preprint (which was not certified by peer review) is the author/funder, who has granted bioRxiv a license to display the preprint in perpetuity. It is made available under aCC-BY 4.0 International license.

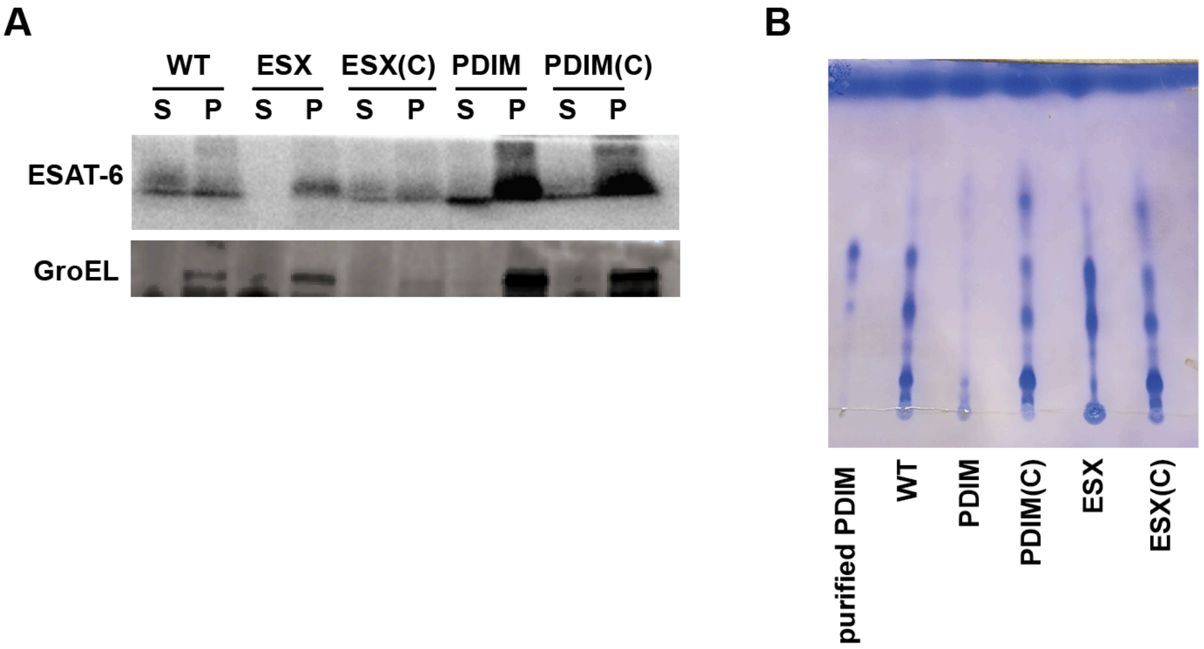

Figure S2: Mtb Erdman PDIM mutants secrete ESAT-6 and ESX-1 mutants produce PDIM

(A) WT, Δ*eccC1* (ESX), complemented Δ*eccC1::eccC1* (ESX(C), *fadD28::tn* (PDIM), or *fadD28::tn* + p*fadD28* (PDIM(C)) *M. tuberculosis* Erdman strain were cultured in Sauton's complete media without Tween-80 for 5 days. Cell lysate and culture supernatant were processed through SDS-PAGE and Western blot analysis. Anti-ESAT-6 antibody was used for detecting ESAT-6. Anti-GroEL antibody was used as a loading control for the supernatant fraction. Results shown are from 1 experiment representative of 2 independent experiments. **(B)** WT, Δ*eccC1* (ESX), complemented Δ*eccC1::eccC1* (ESX(C), *fadD28::tn* (PDIM), or *fadD28::tn* + p*fadD28* (PDIM(C)) *M. tuberculosis* Erdman strain were cultured in 7H9 media. Outer leaflet of mycomembrane was removed via hexanes wash, concentrated and separated on TLC plate with 98:2 petroleum ether:acetone mobile phase. TLC plate was stained with 0.2% Coomassie blue in 20% methanol. Results shown are from 1 experiment representative of 2 independent experiments.

bioRxiv preprint doi: https://doi.org/10.1101/2025.05.08.652811; this version posted May 13, 2025. The copyright holder for this preprint (which was not certified by peer review) is the author/funder, who has granted bioRxiv a license to display the preprint in perpetuity. It is made available under aCC-BY 4.0 International license.

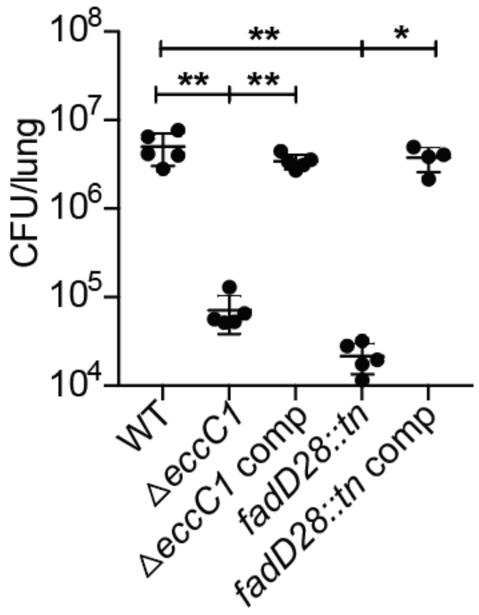

Figure S3. Attenuation of ESX-1 and PDIM mutants at 21 days post infection. WT B6 mice were aerosol infected with either WT, Δ*eccC1,* Δ*eccC1::eccC1*, *fadD28::tn*, or *fadD28::tn* + p*fadD28 M. tuberculosis* Erdman strain. 21 days post infection, mice were sacrificed, and CFU were enumerated from lung lysates. *, $p < 0.05$; **, $p < 0.01$ (unpaired nonparametric Mann-Whitney U test).

bioRxiv preprint doi: https://doi.org/10.1101/2025.05.08.652811; this version posted May 13, 2025. The copyright holder for this preprint (which was not certified by peer review) is the author/funder, who has granted bioRxiv a license to display the preprint in perpetuity. It is made available under aCC-BY 4.0 International license.

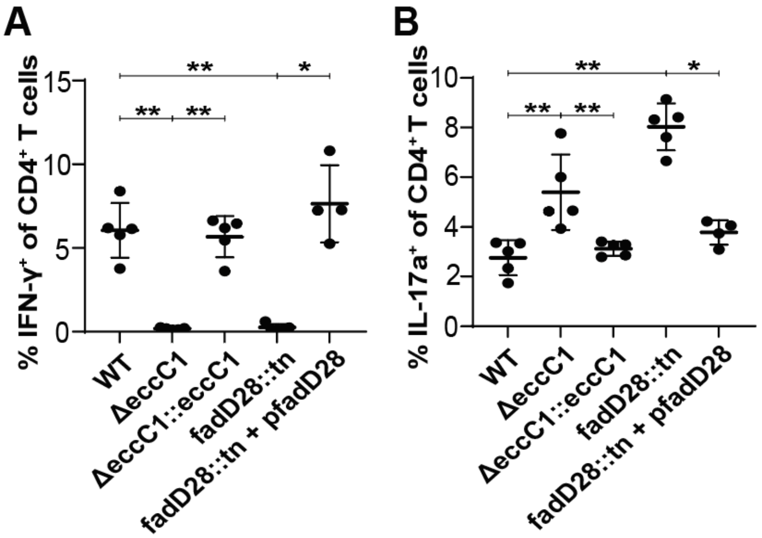

Figure S4: Antigen 85b stim resembles ESAT-6 stimulation for IFN-γ and IL-17a staining of CD4$^+$ T cells. As in Figure 3, WT B6 mice were aerosol infected with either WT, Δ*eccC1,* Δ*eccC1::eccC1*, *fadD28::tn*, or *fadD28::tn* + p*fadD28 M. tuberculosis* Erdman strain. 21 days post infection, mice were sacrificed, lung single cell homogenates were stimulated with Antigen 85b peptide pool and measured for lung **(A)** IFN-γ$^+$ CD4$^+$ T cells and **(B)** IL-17a$^+$ CD4$^+$ T cells by flow cytometry. Results in A and B are representative of 2 independent experiments. *, $p < 0.05$; **, $p < 0.01$ (unpaired nonparametric Mann-Whitney U test

bioRxiv preprint doi: https://doi.org/10.1101/2025.05.08.652811; this version posted May 13, 2025. The copyright holder for this preprint (which was not certified by peer review) is the author/funder, who has granted bioRxiv a license to display the preprint in perpetuity. It is made available under aCC-BY 4.0 International license.

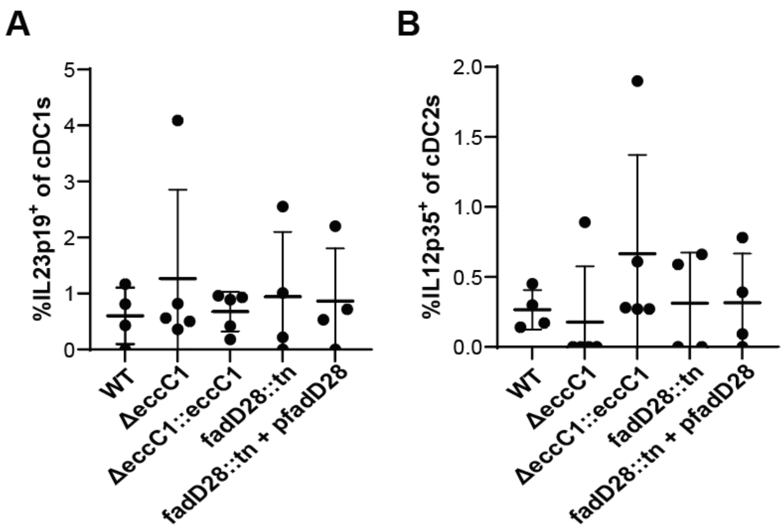

Figure S5: There is little to no IL-23 p19 expression in cDC1s and IL-12 p35 expression in cDC2s during Mtb infection. As in Figure 5, WT B6 mice were aerosol infected with WT, Δ*eccC1,* Δ*eccC1::eccC1*, *fadD28::tn*, or *fadD28::tn* + p*fadD28 M. tuberculosis* Erdman strain. 21 days post infection, mice were sacrificed, mediastinal lymph nodes were extracted, and processed for ICS. Analysis of **(A)** IL-23 $p19^+$ cDC1s and **(B)** IL-12 $p35^+$ cDC2s by flow cytometry. Results in A and B are representative of 2 independent experiments. (unpaired nonparametric Mann-Whitney U test).
